# Differential Proteomic Analysis of Anthers between Cytoplasmic Male Sterile and Maintainer Lines in *Capsicum annuum* L

**DOI:** 10.3390/ijms141122982

**Published:** 2013-11-20

**Authors:** Zhiming Wu, Jiaowen Cheng, Cheng Qin, Zhiqun Hu, Caixia Yin, Kailin Hu

**Affiliations:** 1 College of Horticulture and Landscape Architecture, Zhongkai University of Agriculture and Engineering, Zhongkai Road 501, Guangzhou 510225, Guangdong, China; E-Mails: wuzm2012@gmail.com (Z.W.); caixia0031@163.com (C.Y.); 2 College of Horticulture, South China Agricultural University, Wushan Road 483, Guangzhou 510640, Guangdong, China; E-Mails: gzdragonchan@yeah.net (J.C.); zhqhu@scau.edu.cn (Z.H.); 3 Zunyi Institute of Agricultural Sciences, Zunyi 563102, Guizhou, China; E-Mail: qincheng1001@163.com; 4 Maize Research Institute of Sichuan Agricultural University, Chengdu 611130, Sichuan, China; 5 Key Laboratory of Biology and Genetic Improvement of Maize in Southwest Region, Ministry of Agriculture, Chengdu 611130, Sichuan, China

**Keywords:** *Capsicum annuum* L., cytoplasmic male sterility, anther proteome, two-dimensional gel electrophoresis

## Abstract

Cytoplasmic male sterility (CMS), widely used in the production of hybrid seeds, is a maternally inherited trait resulting in a failure to produce functional pollen. In order to identify some specific proteins associated with CMS in pepper, two-dimensional gel electrophoresis (2-DE) was applied to proteomic analysis of anthers/buds between a CMS line (designated NA3) and its maintainer (designated NB3) in *Capsicum annuum* L. Thirty-three spots showed more than 1.5-fold in either CMS or its maintainer. Based on mass spectrometry, 27 spots representing 23 distinct proteins in these 33 spots were identified. Proteins down-regulated in CMS anthers/buds includes ATP synthase D chain, formate dehydrogenase, alpha-mannosidas, RuBisCO large subunit-binding protein subunit beta, chloroplast manganese stabilizing protein-II, glutathione *S*-transferase, adenosine kinase isoform 1T-like protein, putative DNA repair protein RAD23-4, putative caffeoyl-CoA 3-*O*-methyltransferase, glutamine synthetase (GS), annexin Cap32, glutelin, allene oxide cyclase, *etc*. In CMS anthers/buds, polyphenol oxidase, ATP synthase subunit beta, and actin are up-regulated. It was predicted that male sterility in NA3 might be related to energy metabolism turbulence, excessive ethylene synthesis, and suffocation of starch synthesis. The present study lays a foundation for future investigations of gene functions associated with pollen development and cytoplasmic male sterility, and explores the molecular mechanism of CMS in pepper.

## 1. Introduction

Chili pepper (*Capsicum* spp.) is one of the most widely cultivated vegetables or spice crops around the world. Heterosis in pepper has been shown to increase fruit yield by up to 30% [1]. Currently, the breeding of hybrid seeds depends on hand pollination, which is costly and difficult to ensure seed purity. Selecting male sterile lines, with good combining ability, are an efficient measure to breeders in commercial hybrid seed production.

Cytoplasmic male sterility (CMS) is caused by an interaction between nuclear and mitochondrial genes. Unlike genetic male sterility, CMS belongs to maternal inheritance and is a product of mitochondrial DNA (mtDNA) evolution that gives birth to new genes for better or for worse. Compared with the male fertile mtDNA, those CMS-associated genes are usually some novel open reading frames (ORFs) or chimeric mitochondrial genes, which cause male sterility in the female lines by expressing novel RNA and/or polypeptides [2,3]. In addition, nuclear restorer (*Rf*) genes in the male parent reversely affect the mutant mitochondrial genes and restore pollen viability in their F_1_ hybrid [4–6]. Therefore, this CMS*/Rf* system not only represents a valuable tool in large scale production of commercial hybrid seeds but is also an excellent model to investigate the interaction between nuclear and mitochondrial genomes.

Male sterile cytoplasm of pepper was first documented by Martin and Grawford (1951) and characterized by Peterson (accession USDA P.I. 164835) [7,8], which is the only known source of male sterile cytoplasm until now. In order to explore the molecular mechanism of pepper CMS, *orf456* or *orf507* had been identified as a candidate gene [9,10]. *Orf456* contains a novel ORF at the 3′-end of the *cox* II gene, which is co-transcribed with the *cox* II gene encoding a subunit of cytochrome c oxidase in mitochondria and inducing male sterility in *Arabidopsis thaliana* [9]. Gulyas found that a cytosine (C) deletion at the original stop codon of *orf456* leads to continue to extend its translation to a new termination codon downstream, and finally produces a total length of 507 bp RNA, named *orf507* [10]. However, they did not reveal the full mechanism of the CMS phenomenon in chili pepper. Recently, Luo *et al*. (2013) had identified the molecular basis of male sterility in the rice CMS-WA system. They thought that a new mitochondrial gene, *WA532*, accumulates preferentially in the anther tapetum, thereby inhibiting COX11 function in peroxide metabolism and triggering premature tapetal programmed cell death (PCD) and consequent pollen abortion [6].

As the functional molecules of living cells, proteins play important roles in the development of cell, organ or tissue by the interaction with other molecules or modifications. To study their functions, proteomics is one of the most powerful molecular tools in describing the proteomes of different organelles or tissues [11–13]. In recent years, the proteomic approach has been also applied to explore anther development and pollen reproduction of plants, such as in rice [11], tomato [12], *Brassica napus* [13], wolfberry [14], tobacco [15], *etc*. However, there is little proteomic information available for answering the unresolved mysteries and clarifying the mechanism of CMS in pepper.

In our study, a comparative proteomic approach was used to identify differentially expressed proteins of anthers during reproductive development of CMS in cytoplasmic male sterile and maintainer Lines in *Capsicum annuum* L. The objective was to identify some specific proteins related to CMS, and further elucidate their possible biological roles and their potential effects on anther development and pollen fertility. This lays a foundation for the understanding of molecular mechanism of CMS at the proteomic level, and makes the better use of CMS/*Rf* system in the production of pepper hybrid seeds.

## 2. Results

### 2.1. Abundance Analysis of Differentially Expressed Proteins in the Normal and Sterile Cytoplasm

**Figure 1:**
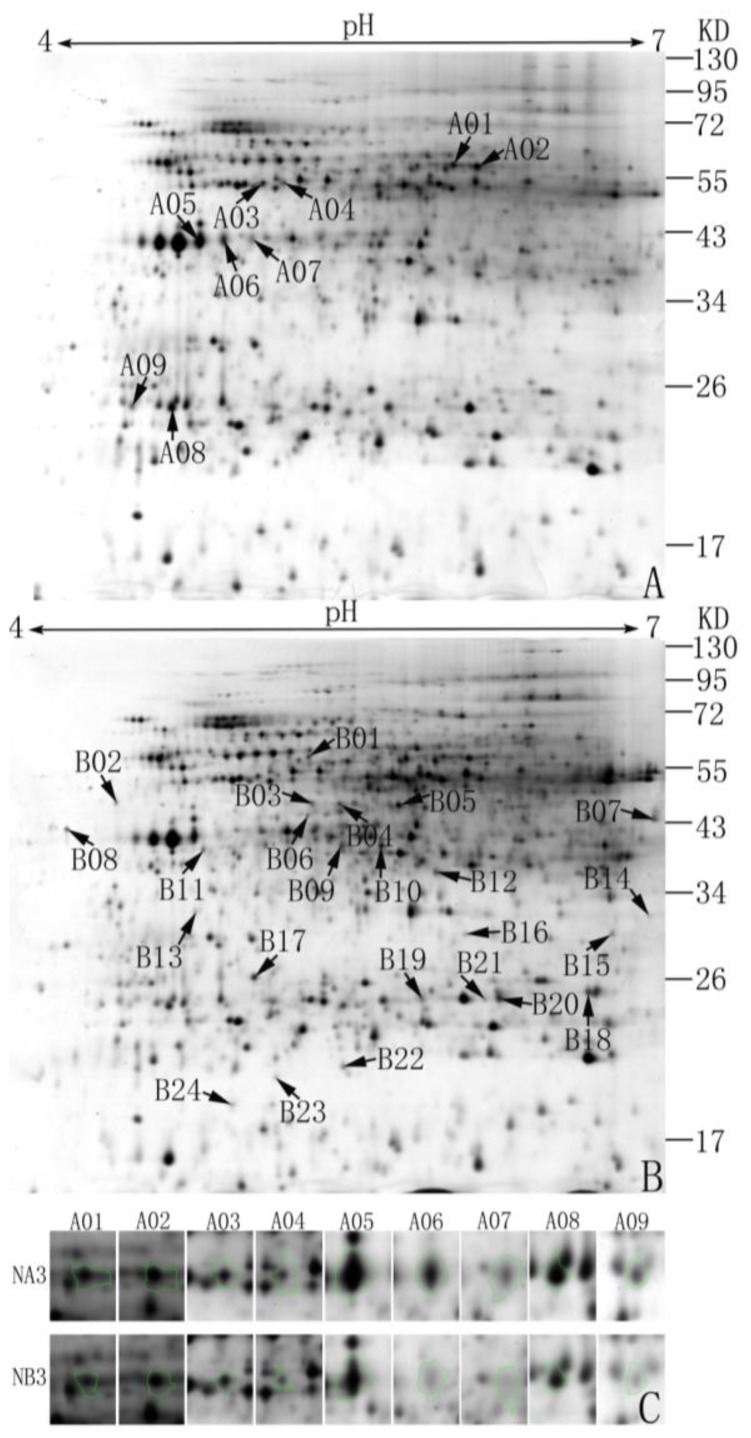
Two-dimension electrophoresis gels of anther/bud proteins in CMS NA3 line (**A**) and its maintainer NB3 line (**B**); some of the areas in NA3 and NB3 gels with differential spots have been enlarged below (**C**). Proteins were visualized by silver nitrate staining. The arrowed and numbered spots in the image were differentially expressed proteins. Molecular markers (kDa) are shown on the right. These results were repeated for three times.

The objective of this study was to identify differentially expressed proteins between sterile and fertile anthers (*i.e*., NA3 and NB3 isogenic lines). Proteins were firstly separated by the isoelectric focusing (IEF) on a linear gradient ranging from pH 4.0 to 7.0. Based on the size of molecular masses, proteins were separated by SDS-PAGE and then stained with CBB G250 and silver nitrate (Figure 1A,B). As shown in Figure 1C, some differential spots were found in the enlarged portion of gels. Only proteins detected in all two reps were recorded. Over 1200 non-redundant proteins were found in the anther proteome. By contrast, 33 different proteins were determined between A and B lines according to their volume ratios (more than 1.5-fold, *p* ≤ 0.05). Proteins with less than 1.5-fold change in volume were not considered in this study. Of these, nine proteins had much higher concentrations in sterile anthers/buds (Figure 1A), 11 proteins had lower concentrations in sterile anthers/buds, and 13 proteins were not translated in sterile anthers/buds (Figure 1B).

### 2.2. Identification and Functional Classification of Differentially Expressed Proteins

Thirty-three different proteins detected were excised from the preparative gels and digested with trypsin. Of these, 27 protein spots (81.82%) were successfully identified by MALDI-TOF/TOF MS analysis except for A5, A6, A8, A9, B8, and B18. Compared with NCBInr and pepper ESTdatabases, 23 distinct proteins identified were noted with ID, MW, pI value, MASCOT score, number of matched peptides, and sequence coverage ratio (%) (Tables 1, S1). In male sterile cytoplasm, A01 and A02 were identified as Polyphenol oxidase F, and ATP synthase subunit beta was showed in A03 and A04, In normal cytoplasm, multiple spots were also detected, such as ATP synthase CF1 alpha chain (B03 and B04), Glutelin (B14 and B15), glutamine synthetase (B09, B10, and B12), Actin (B06 and A07), and triose phosphate isomerase cytosolic isoform (B19 and B21). These results indicated that some proteins species with post-translational modifications may play a certain role in multiple organelles such as mitochondria, plastids, and cystol.

In male sterile anthers/buds, higher expression proteins related to fertile anthers/buds include polyphenol oxidase F, ATP synthase subunit beta, and actin. While the down-regulated proteins have ATP synthase D chain, formate dehydrogenase, alpha-mannosidas, RuBisCO large subunit-binding protein subunit beta, chloroplast manganese stabilizing protein-II, glutathione *S*-transferase, adenosine kinase isoform 1T-like protein, putative DNA repair protein RAD23-4, and putative caffeoyl-CoA 3-*O*-methyl transferase. Proteins only showed in fertile anthers/buds contain ATP synthase CF1 alpha chain, triose phosphate isomerase (TPI), 23 kDa polypeptide of the oxygen evolving complex of photosystem II, glutamine synthetase (GS), annexin Cap32, glutelin, and allene oxide cyclase.

Based on their putative physiological functions compared with the gene annotations in NCBI and some relevant literatures, 27 identified proteins are classified into nine functional groups, such as respiration and energy pathway, carbohydrate metabolism, antioxidative reactions, photosynthesis, amino acid metabolism, singal transduction, cytoskeleton, storage protein, and others. The largest functional category of proteins drop into respiration and energy pathway (22.22%, two in sterile anthers and four in fertile anthers), followed by those proteins participating in carbohydrate metabolism (11.11%, three only expressed in maintainer anthers), photosynthesis (11.11%, three only expressed in maintainer anthers), antioxidative reactions (11.11%, two in CMS and one in maintainer anthers), and amino acid metabolism (11.11%, three only expressed in maintainer anthers).

More differentially expressed proteins were in fertile anthers/buds than in sterile anthers/buds. Polyphenol oxidase F (PPO, showed in A01 and A02 spots, Tables 1, S1) was a major protein showed significantly up-regulation in male sterile cytoplasm. Therefore, this enzyme was selected to further confirm changes using enzyme activities.

### 2.3. Evaluation of PPO and EFE Activities

**Figure 2:**
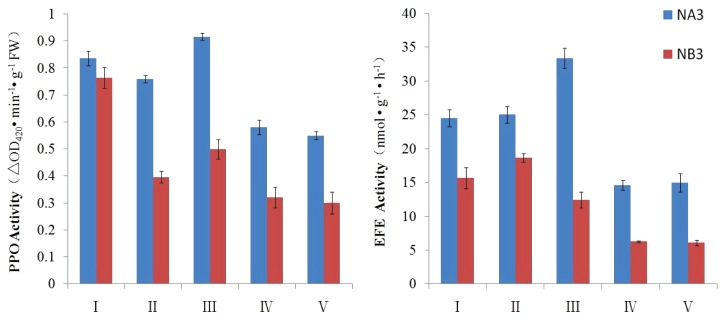
The activities of PPO and EFE in CMS and its maintainer line during different development stages. Error bars indicate standard deviation. Roman letters I to V marked represent the five stages of flower buds indicated in Figure 3F.

The activity of PPO and ethylene forming enzyme (EFE) enzymes in CMS and maintainer line were analyzed at different stages of pollen development. As showed in Figure 2, the activities of PPO and EFE are always higher in CMS line NA3 than in maintainer line NB3. Especially, in the stage II and stage III, the activity of PPO was nearly two times higher in CMS compared with that in maintainer line (Figure 2). In stage III, the activity of EFE was nearly three times higher in CMS than that in maintainer line.

## 3. Discussion

The use of CMS/*Rf* system has been widely applied to F_1_ hybrid seed production of chili pepper. However, the molecular mechanism of CMS remains poorly understood. As a powerful molecular tool, proteomics has been used to study anther development and pollen reproduction in other plants [11–15]. In our study, the proteomic approach was performed to identify differentially expression proteins in CMS and its maintainer line to understand the molecular mechanism of pepper male sterility. Thirty-three proteins displayed differential expression levels between male sterile and normal anthers/buds. These down-regulated proteins identified using MS in CMS anthers/buds included RuBisCO large subunit-binding protein subunit beta, ATP synthase CF1 alpha chain, alpha-mannosidas, formate dehydrogenase, glutamine synthetase, caffeoyl-CoA 3-*O*-methyltransferase, triose phosphate isomerase, and glutathione *S*-transferase. The other proteins up-regulated in male sterile anthers/buds include polyphenol oxidase, ATP synthase subunit beta, and actin. The potential roles of some differential expression proteins in anther and pollen development are discussed as following.

### 3.1. Proteins Involved in Respiration and Energy Pathway

Six identified proteins belong to these groups of respiration and energy pathways. In particular, ATP synthase CF1 alpha chain (B03), ATP synthase CF1 alpha subunit (B04), formate dehydrogenase (B07), and ATP synthase D chain (B24) were down-regulated in the CMS line. In CMS lines of rice, *Brassica napus*, and wolfberry, two enzymes are also involved in respiration and energy metabolism [11,13,14].

Anther and pollen development is a complex process that depends on a series of well-coordinated metabolic and structural changes. During flower development, male meiotic cells and developing microspores require higher energy, particularly much higher mitochondrial biosynthetic products than vegetative development and growth. In maize, the number of mitochondria per cell increases 20- and 40-fold in meiocytes and tapetal cells during pollen development [16]. Microspore death was observed, when antisense of mitochondrial pyruvate dehydrogenase El down-regulated the alternative oxidase by suppressing pyruvate dehydrogenase in tobacco tapetum [17]. Many CMS-associated genes include portions of ATP synthase subunits or closely linked to normal ATP synthase subunit genes. The ATP synthase, a key enzyme for the synthesis of ATP for cellular biosynthesis, comprises three parts: F0, F1, and FA [18,19]. It has been reported that alterations of mitochondrial-encoded subunits of the F_0_F_1_-ATP synthase induce CMS in plants. For instance, in sunflower, sterile plants expressing mitochondrial ORF522 showed a specific decreased ATP synthase activity [20]. The chimeric mitochondrial ORF522 shares sequence similarity with ORFB, a plant-type ATP8, which might result in competition between two proteins leading to decreased activity of the F_0_F_1_-ATP synthase complex. In CMS-HongLian rice, sterility is associated with the expression of *atp6-OrfH79*, which might disturb the formation of the F_0_F_1_-ATPase complex, resulting in decreased activity of ATPase and pollen abortion [21]. The *Ψatp6-2* in the pepper CMS line HW203A was also down-regulated, but it was up-regulated in the maintainer line. The corresponding F_0_F_1_-ATPase activity in the CMS line was gradually decreased along with the development of the anther, while maintainer line, F_0_F_1_-ATPase activity sharply decreased after the stage of sporogenous cell, but gradually increased following the tetrad stage [22]. One hypothesis on the mechanism of CMS is that the sterile lines are unable to meet the increased energy demand in the form of ATP during anther development, that leading to pollen abortion [20,23].

### 3.2. Proteins Involved in Carbohydrate Metabolic Pathways

Carbohydrate metabolism is one of the most basic metabolic pathways in biological metabolism. Its main physiological function is to provide required energy and carbon sources. Two proteins expressed only in NB3 anthers that were involved in carbohydrate metabolism were triose phosphate isomerase (TPI, B19, and B21) and alpha-mannosidase (B05). TPI plays an important role in the glycolytic pathway, which is the glycolytic enzyme. It can catalyze the reversible inter conversion of glyceraldehyde 3-phosphate and dihydroxyacetone phosphate [24]. Moreover, glycolysis belongs to the common method of aerobic and anaerobic respiration, which is critical for energy supply.

Carbohydrate plays a key role in anther and pollen development. It not only provides nutrition for anther development, but also affects anther and pollen development as a signal substance. Deng reported that the content of ATP and NADH decreased significantly in the maintainer line at the stage of pollen abortion, meanwhile, the expression level of TPI reduced obviously in anther [25]. When restorer genes (*Rf*s) were crossed to sterile lines, TPI activity and the expression level were increased and maintained at the normal levels. Therefore, we conclude that decreased abundance of these enzymes in CMS anthers down regulated sugar and starch concentrations, two molecules are necessary for biosynthesis and energy balance and induce male sterility.

### 3.3. Proteins Involved in Photosynthesis

Three spots (B01, B13, and B23) showed higher concentrations in fertile anthers/buds. They were identified as chloroplastic RuBisCO large subunit-binding protein subunit beta, chloroplast manganese stabilizing protein-II, and 23 kDa polypeptide of the oxygen evolving complex of PSII. Photosynthesis is dependent on the coordinated activity of the chloroplast, mitochondrial, and nuclear components [26]. Photosynthesis provides some substrates for mitochondrial respiration but it also depends on several compounds synthesized by mitochondria. In the dark, mitochondria are the main source of ATP for cellular processes, including those in the chloroplasts. Moreover, the mitochondrial ATP maintains the proton gradient across the thylakoid membrane, thus protecting the chloroplast from photo inhibition upon reillumination [27]. In the light, mitochondria provide the chloroplast with carbon skeletons for NH_4_^+^ assimilation [28], while ATP supports various biosynthetic reactions, including the repair and recovery of photosystem II (PSII). The tobacco (*Nicotianata bacum*) CMSII mutant lacks the major mitochondrial NADH dehydrogenase (Complex I) and exhibits a decrease in the rate of photosynthesis, notably during dark-light transitions or when carbon fixation and photo respiration are simultaneously active [29,30]. It is possible that, due to a lack of these photosynthetic proteins, in the CMS line of pepper, a decrease in the rate of photosynthesis might lead to pollen abortion.

### 3.4. Proteins Involved in Antioxidative Reactions

**Figure 3:**
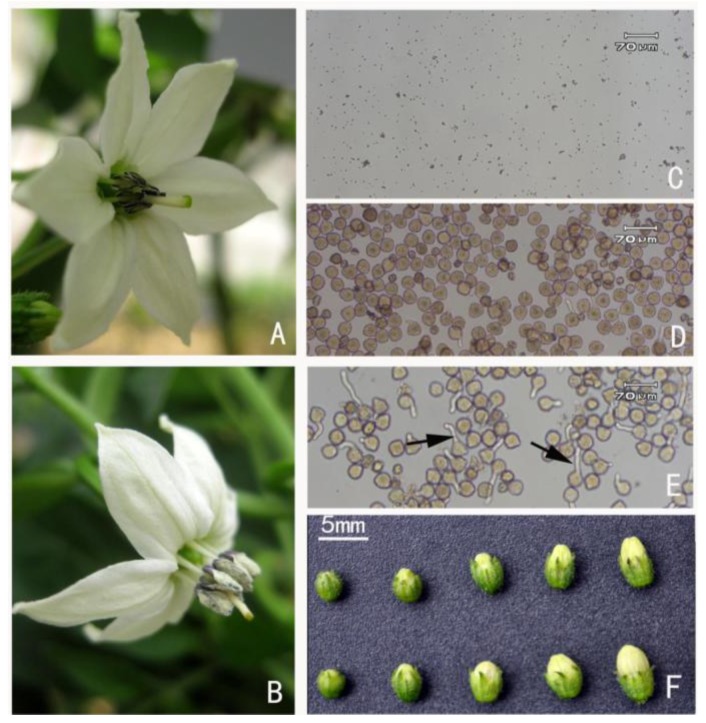
Morphological changes in flower organs in A & B lines. (**A**) Mature sterile A-line anthers are brown and senescence; (**B**) Mature fertile B-line anthers are plump and dehiscent; (**C**) No pollen present in NA3 anthers; (**D**) Pollen was present in NB3 anthers; (**E**) Germination of NB3 pollen; (**F**) Flower buds of five different development stages. In addition to stage I and II, using whole buds, anthers in other stages were used as materials in this experiment. Top row: NA3. Bottom row: NB3.

PPO is a nuclear encoded gene whose enzyme attaches to the thylakoids of chloroplasts [31]. It can be released during ripening or senescence and associated with browning in fruits and vegetables like bananas, avocados, cocoa, and tea. In our study, two spots (A01 and A02) were identified as polyphenol oxidase (PPO), which showed much higher concentrations in sterile anthers/buds. As shown in Figure 3A, anthers in male sterile become gradually brown and senescence. Therefore, we would expect to find an abundance of this enzyme in sterile anthers. These results are consistent with premature cell death and point to the A-gene’s inhibition of the cox II enzyme, which is known to cause premature tapetal programed cell death (PCD). The up-regulation of PPO in sterile anthers is a symptom of CMS. This result supports the inhibition of the cox II enzyme in our A-B molecular model.

### 3.5. Proteins Involved in Amino Acid Metabolism

One of the most notable differences in NA3 and NB3 anther/buds gels was the location of glutamine synthetase protein (GS, B09, B10, and B12). As these enzymes encoded by nuclear genes, they are not candidates for causes of cytoplasmic male sterility. In addition, they are targeted to chloroplast and cystosol, so the multiple spots were detected in the gel [32].

Other anther proteins with higher spot volumes in the maintainer line might be involved in signal transduction such as adenosine kinase isoform 1T-like protein (B11) and annexin Cap32 (B16). Glutelin (B14, B15) belongs to storage protein. Others, like RAD23-4(B02), putative caffeoyl-CoA 3-*O*-methyltransferase (B17), and allene oxide cyclase (B22), are considered as putative DNA repair proteins (Tables 1, S1). Adenylate kinase (AK, E.C. 2.7.4.3) is a phosphotransferase enzyme that catalyzes the interconversion of adenine nucleotides, and plays an important role in cellular energy homeostasis and transfer. In Arabidopsis, AK deficiency lead to fertility decrease and the stamen filaments do not elongate normally [33].

Annexins interact in a calcium-dependent manner with membrane phospholipids, and have been proposed to be involved in a variety of cellular processes. In cabbage (*Brassica oleracea* L. var. *capitata* L.), *BoAnnexin*2 gene plays an important role in pollen germination [34]. Allene oxide cyclase (AOC) catalyzes the stereospecific cyclization of unstable alleneoxideto *cis-*(+) 12-oxo-phytodienoicacid (OPDA), which is a crucial step in the biosynthsis of jasmonic acid (JA). The AOC protein accumulated in ovules and in parenchymatic cells of vascular bundles of flower stalks [35]. JA and *cis*-(+)-OPDA are not only important signaling molecules in the coordination of plant response, but also play an important role in the regulation of developmental processes [36]. In *Arabidopsis thaliana*, JA is important for the release of pollen and elongation of filaments. JA-deficient or JA-insensitive plants are male sterile. The similar results had been reported in the moss *Physcomitrella patens* [37].

## 4. Experimental Section

### 4.1. Plant Materials and Anther Collection

The pepper CMS line NA3 and its maintainer line NB3 with the same nuclear background were used in this study. NA3 with stable male sterile was generated by backcrossing the male sterile line of 8907A [38] as the genetic background of sterile cytoplasm, and the common chili pepper inbred line, North3, as the male parent. The backcrossing has been over 15 generations. Therefore, any differences in anther proteins between these two lines are attributed to their cytoplasms. Plants were field grown without nutrient and moisture stress at the Zengchen Experimental Station, South China Agricultural University, Guangzhou, China (23°8N, 113°17E). Flower buds were cut from NA3 and NB3 at five temporal stages that targeted phase 1 of anther development (Figure 3F). Bulk samples were collected from 30 individuals. The whole flower buds of stage I and II, and anthers of other stages, were excised and immediately frozen in liquid nitrogen. Following, the samples are stored at −80 °C until protein extraction.

### 4.2. Protein Extraction and 2-DE Electrophoresis

Protein extractions were performed using a trichlor-oacetic acid (TCA)-acetone protocol with some modifications [13]. The samples were ground to a fine powder in liquid nitrogen using a pestle and mortar. The samples were transferred to a centrifuge tube and immediately suspended in 10 volumes of TCA/acetone solution (10% *w*/*v* TCA and 0.07% *w*/*v* 2-mercaptoethanol in acetone) for over-night protein precipitation at −20 °C to remove secondary metabolites. Samples were centrifuged at 4 °C for 15 min at 13,000× *g*, and precipitated proteins were washed twice with three volumes of cold (−20 °C) 100% acetone, incubated at −20 °C for 2 h, and then centrifuged. The pellet was air-dried and resuspended in solubilization buffer comprising 7 M urea, 2 M thiourea, 4% CHAPS, and 30 mM Tris, adjusted to pH 8.5. The solution was incubated at 4 °C for 1 h with continuous shaking, and then centrifuged at 4 °C for 15 min at 13,000 × *g*. The supernatant was collected and stored at −80 °C for further analysis. Protein concentrations were determined by Bradford assay (Bio-Rad, Alfred Nobel Drive Hercules, CA, USA) with BSA as the standard.

Isoelectric focusing (IEF) was carried out using an IPGphor III electrophoresis system (GE Healthcare, Pittsburgh, PA, USA) and 24 cm immobiline dry strips, pH 4–7 (GE Healthcare, Pittsburgh, PA, USA). Nine hundred micrograms of protein samples was loaded during the rehydration step (14 h). IEF was then performed by ramping to 300 V for 30 min, at 700 V for 30 min, and 1500 V for 1.5 h successively, ramping to 9000 V over 1 h, and holding at 9000 V, until a total of 52 kV was reached. Prior to the second dimension separation, the IPG strips were equilibrated in equilibration buffer (6 M urea, 30% (*w*/*v*) glycerol, 2% (*w*/*v*) SDS, 50 mM Tris-HCl, pH 8.0), first with 1% DTT and then with 2.5% iodoacetamide each for 15 min. The electrophoresed and equilibrated strips were then transferred to 12.5% vertical SDS-PAGE gels for the second dimension electrophoresis using an EttanDALT six Large Vertical System (GE Healthcare, Pittsburgh, PA, USA). SDS-PAGE was run at 2 W/gel for 45 min and then 17 W/gel until the bromphenol blue dye front reached the gel end. After electrophoresis, gels were stained with Coomassie Brilliant Blue (CBB) G-250 and silver nitrate.

### 4.3. Gel Imaging and Analysis

For image acquisition, the stained gels were scanned using ImageScanner III (GE Healthcare, Pittsburgh, PA, USA) at a resolution of 300 dpi and 16-bit grayscale pixel depth. The comparison between NA3 and NB3 gels was made with Image Master 2D Platinum software, Version 5.0 (GE Healthcare, Pittsburgh, PA, USA) as described in the user manual. Background subtraction and normalization were fully automatic. Minimal manual editing was performed to correct mismatched and unmatched spots between gels. The average vol.% values were calculated from three technical replicates to represent the final vol.% values of each biological replicate. NA3 and NB3 spots with 1.5-fold change or more and *p* < 0.05 were considered to be differentially expressed.

### 4.4. Trypsindigestion, Mass Spectrometry, and Protein Identification

The selected spots were excised from 2-D gels, and pellets were washed with 100 μL destaining solution (50 mM NH_4_HCO_3_ in 50% ACN) in micro centrifuge tubes. This step was repeated for two to three times until the pellets were colorless. The gel pieces were washed by Milli-Q water and lyophilized, then rehydrated in digestion buffer containing 20 μg/mL of sequencing grade modified trypsin (Promega, Madison, WI, USA) in 25 mM NH_4_HCO_3_ at 37 °C overnight. After brief centrifugation, the peptides were collected from supernatant, and left gel pieces were further sonicated for 10 min in 5 μL of 0.1% TFA (trifluoroacetic acid) in 50% ACN to collect the remaining peptides. The peptides from one protein spot were combined.

Mass spectrometry analysis was carried out using Autoflex speed™ MALDI-TOF-TOF (Bruker Daltonics, Bremen, Germany) tandem mass spectrometer. Parameters were set as follows: laser intensity 4500, mass range 700-3, 200 kDa, acceleration voltage 20 kV, repeat rate 200 Hz. Peptide mass finger printings (PMFs) obtained from MAIDI-TOF/MS were used to search the NCBInr protein database (http://www.ncbi.nlm.nih.gov/) and SwissProt protein databases using the MASCOT program (http://www.matrixscience.com). The following search parameters were used: monoisotopic peptide mass; 800–4000 Da; one missed cleavage per peptide; enzyme, trypsin; taxonomy, green plants; precursorion mass tolerance, 50 ppm; MS/MS fragmention mass tolerance, 0.5 Da; variable modifications, carbamidom ethylation for cysteine, and oxidation for methionine were allowed. Known contaminant ions corresponding to trypsin and keratins were excluded from the peak lists before database searching. Top six hits for each protein search were reported. Only proteins with Mascot proteins cores (based on both MS and MS/MS spectra) of 80 or more (*p* < 0.05), and a minimum of two matched peptides were considered to be positively identified.

### 4.5. PPO and EFE Activity Assay

Polyphenol oxidase (PPO) activity was determined according to the method of Leja *et al*. [39]. One milliliter reaction mixture contained 20 μL enzyme extract and 10 mmol/L phosphate buffer (pH 7.0). Each sample was aerated for 2 min in a small test tube followed by the addition of catechol as the substrate at a final concentration of 20 mmol/L. PPO activity was presented as the change in one unit of absorbance at 420 nm per minute per gram fresh weight of sample.

Ethylene forming enzyme (EFE) activity was determined according to the method of Wei and Kenji [40]. Zero point three grams of the buds or anthers was sealed in a vial with 2.5 mL reaction buffer (pH 7.2), 1 mmol/L ACC, 100 mmol/L MOPS. After 20 min of continuous shaking at 35 °C, 1 mL gas sample of the vial atmosphere was withdrawn by syringe and its ethylene content was determined by gas chromatography (GC-17A, SHIMADZU, Nakagyo-ku, Kyoto, Japan).

## 5. Conclusions

In summary, a comparative proteomic approach was used to identify differentially expressed proteins in developing anthers of CMS and its maintainer of *Capsicum annuum* L. Two-dimensional gel electrophoresis (2-DE) was used to identify 27 proteins that showed more than 1.5-fold volume ratio difference in the two lines. The results showed the breakdown of pollen development in the CMS line was associated with differential expression of certain proteins. We speculated that male sterility in NA3 might be related to energy metabolism turbulence, excessive ethylene synthesis or suffocation of starch synthesis. Therefore, this study lays the foundation for future investigation of gene function related to pollen development and cytoplasmic male sterility in pepper.

## Figures and Tables

**Table 1:** Summary of differentially expressed proteins identified by mass spectrometry analysis.

Spot No. a	Protein name (Species) b	Accession No. c	kDa/pI (Theor d)	kDa/pI (Exper e)	Mascot score	Peptides matched	S.C. (%) f	A/Bspot volume ratio g
**Respiration and energy pathway (6)**								

A03	ATP synthase subunit beta (NP)	gi|114421	59.93/5.95	53.95/5.10	370	13	33	+1.69
A04	ATP synthase subunit beta (NP)	gi|114421	59.93/5.95	54.05/5.18	362	13	35	+3.09
B03	ATP synthase CF1 alpha chain (SL)	gi|89280620	55.43/5.14	48.34/5.30	453	16	30	−∞
B04	ATP synthase CF1 alpha subunit (NS)	gi|78102516	55.44/5.14	47.25/5.43	549	15	27	−∞
B24	ATP synthase D chain (SD)	gi|48209968	19.80/5.34	19.21/5.00	266	13	55	−13.53
B07	Formate dehydrogenase, mitochondrial (ST)	gi|26454627	42.30/6.64	34.00/6.50	202	10	33	−1.73

**Carbohydrate metabolism (3)**								

B05	alpha-mannosidase (CA)	gi|315440801	47.03/6.28	47.09/5.67	284	14	12	−4.01
B19	triose phosphate isomerase cytosolic isoform (SC)	gi|38112662	27.25/5.13	24.97/5.74	279	6	21	−∞
B21	triose phosphate isomerase cytosolic isoform (SC)	gi|38112662	27.25/5.73	24.74/5.98	358	5	27	−1.60

**Photosynthesis (3)**								

B01	RuBisCO large subunit-binding protein subunit beta, chloroplastic RuBisCO (VV)	gi|225442531	65.26/5.62	58.16/5.30	128	4	8	−1.60
B13	chloroplast manganese stabilizing protein-II (ST)	gi|239911810	31.44/6.45	32.74/5.00	284	4	16	−1.61
B23	23 kDa polypeptide of the oxygen evolving complex of photosystem II (SA)	gi|241865142	22.91/5.14	20.41/5.16	131	2	9	−∞

**Antioxidative reactions(3)**								

A01	Polyphenol oxidase F (SL)	gi|1172583	66.77/6.27	58.38/5.85	218	6	8	+1.76
A02	Polyphenol oxidase F (SL)	gi|1172583	66.77/6.27	57.93/5.94	212	8	10	+1.53
B20	glutathione S-transferase (AH)	gi|254798560	24.86/6.96	24.67/6.03	83	2	9	−2.76

**Amino acid metabolism (3)**								

B09	glutamine synthetase(SL)	gi|541632	18.74/6.12	34.00/5.44	274	4	15	−∞
B10	glutamine synthetase (AM)	gi|12802875	39.43/6.17	34.00/5.59	305	6	22	−∞
B12	glutamine synthetase (SL)	gi|541632	18.74/6.12	34.00/5.80	128	5	15	−∞

**Signal transduction (2)**								

B11	adenosine kinase isoform 1T-like protein (ST)	gi|82400168	39.75/5.01	34.00/5.00	81	3	10	−2.89
B16	annexin Cap32(CA)	gi|3979715	35.95/5.85	29.85/5.91	530	20	55	−∞

**Cytoskeleton (2)**								

A07	Actin(NA)	gi|378724806	27.95/5.20	34.00/5.05	279	8	44	+2.27
B06	Actin (NA)	gi|378724806	27.95/5.20	47.00/5.29	118	8	44	−∞

**Storage protein (2)**								

B14	Glutelin (OS)	gi|225710	56.74/8.93	31.20/6.50	92	5	10	−6.47
B15	Glutelin (OS)	gi|20217	56.45/9.17	29.23/6.50	158	6	16	−∞

**Others (3)**								

B02	putative DNA repair protein RAD23-4 (AT)	gi|145334669	34.81/4.85	48.84/5.00	83	2	5	−2.22
B17	putative caffeoyl-CoA 3-O-methyltransferase(CA)	gi|193290676	27.92/5.30	26.07/5.07	231	9	36	−2.84
B22	allene oxide cyclase (NT)	gi|40644130	26.68/6.07	20.86/5.42	88	2	7	−∞

a Spot No. in 2-DE gel, as shown in Figure 1. Where A and B represent sterile and normal cytoplasm, respectively;

b Protein names and species from the NCBInr/EST database. *Nicotiana plumbaginifolia* (NP), *Solanum lycopersicum* (SL), *Nicotiana sylvestris* (NS), *Solanum demissum*(SD), *Solanum tuberosum* (ST), *Sonneratia alba* (SA), *Capsicum annuum*(CA), *Arachis hypogaea* (AH), *Avicennia marina* (AM), *Solanum chacoense*(SC), *Vitis vinifera* (VV), *Neosinocalamus affinis* (NA), *Oryza sativa* (OS), *Arabidopsis thaliana* (AT), *Nicotiana tabacum*(NT);

c Accession number in NCBInr/EST database;

d Theoretical molecular weight and pI of the identified proteins;

e Expect molecular weight and pI of the identified proteins;

f Sequence Coverage;

g The ratios of spots volume are the average for each spot from three replicate gels where “+” denotes multiple in sterile anthers and “−” denotes multiple in fertile anthers.

## References

[1] Guo J.Z., Ma J.H. (1984). Determination of the combining ability of several quantitative traits constituting yield. China Veg.

[2] Hanson M.R., Bentolila S. (2004). Interaction of mitochondria and nuclear genes that affect male gametophyte development. Plant Cell.

[3] Schnable P.S., Wise R.P. (1998). The molecular basis of cytoplasmic male sterility and fertility restoration. Trends Plant Sci.

[4] Wang Z.H., Zou Y.J., Li X., Zhang Q., Chen L., Wu H., Su D., Chen Y., Guo J., Luo D. (2006). Cytoplasmic male sterility of rice with boro II cytoplasm is caused by a cytotoxic peptide and is restored by two related PPR motif genes via distinct modes of mRNA silencing. Plant Cell.

[5] Hu J., Wang K., Huang W., Liu G., Gao Y., Wang J., Huang Q., Ji Y., Qin X., Wan L. (2012). The rice pentatricopeptide repeat protein *RF* 5 restores fertility in Hong-Lian cytoplasmic male-sterile lines via a complex with the glycine-rich protein GRP162. Plant Cell.

[6] Luo D.P., Xu H., Liu Z.L., Guo J.X., Li H.Y., Chen L.T., Fang C., Zhang Q.Y., Bai M., Yao N. (2013). A detrimental mitochondrial-nuclear interaction causes cytoplasmic male sterility in rice. Nat. Genet.

[7] Martin J., Grawford J.H. (1951). Several types of sterility in *Capsicum frutescens*. Proc. Am. Soc. Hortic. Sci.

[8] Peterson P.A. (1958). Cytoplasmically inherited male sterility in *Capsicum*. Am. Nat.

[9] Kim D.H., Kang J.G., Kim B.D. (2007). Isolation and characterization of the cytoplasmic male sterility-associated *orf456* gene of chili pepper (*Capsicum annuum* L.). Plant Mol. Biol.

[10] Gulyas G., Shin Y., Kim H., Lee J.S., Hirata Y. (2010). Altered transcript reveals an *orf507* sterility-related gene in chili pepper (*Capsicum annuum* L.). Plant Mol. Biol. Rep.

[11] Wen L., Liu G., Li S.Q., Wan C.X., Tao J., Xu K.Y., Zhang Z.J., Zhu Y.G. (2007). Proteomic analysis of anthers from Honglian cytoplasmic male sterility line rice and its corresponding maintainer and hybrid. Bot. Stud.

[12] Sheoran I.S., Ross A.R., Olson D.J., Sawhney V.K. (2009). Differential expression of proteins in the wild type and 7B-1 male-sterile mutant anthers of tomato (*Solanum lycopersicum*): A proteomic analysis. J. Proteomics.

[13] Sheoran I.S., Sawhneya V.K. (2010). Proteome analysis of the normal and Ogura (*ogu*) CMS anthers of *Brassica napus* to identify proteins associated with male sterility. Botany.

[14] Zheng R., Yue S., Xu X., Liu J., Xu Q., Wang X.L., Han L., Yu D.Y. (2012). Proteome analysis of the wild and YX-1 male sterile mutant anthers of wolfberry (*Lycium barbarum* L.). PLoS One.

[15] Qi J.M., Ma H.B., Xu J.T., Chen M.X., Zhou D.X., Wang T., Chen S.H. (2012). Proteomic analysis of bud differentiation between cytoplasmic male-sterile line and maintainer in tobacco. Acta Agron. Sin.

[16] Lee S.J., Warmke H.E. (1979). Organelle size and number in fertile and T-cytoplasmic male-sterile corn. Am. J. Bot.

[17] Yui R., Iketani S., Mikami T., Kubo T. (2003). Antisense inhibition of mitochondrial pyruvate dehydrogenase subunit in anther tapetum causes male sterility. Plant J.

[18] Siedow J.N., Umbach A.L. (1995). Plant mitochondrial electron transfer and molecular biology. Plant Cell.

[19] Xu K., Cao M.J., Zhu Y.G., Pan G.T., Rong T.Z. (2008). Differential expression of mitochondrial proteins between C-type cytoplasmic male sterility line C48-2 and its maintainer line in maize. Acta Agron. Sin.

[20] Sabar M., Gagliardi D. (2003). ORFB is a subunit of F_1_F_0_-ATPsynthase: In sight into the basis of cytoplasmic male sterility in sunflower. EMBO Rep.

[21] Zhang H., Li S.Q., Yi P., Wan C.X. (2007). A honglian CMS line of rice displays aberrant F_0_ of F_1_F_0_-ATPase. Plant Cell Rep.

[22] Ji J.J., Huang W., Yin C.C., Gong Z.H. (2013). Mitochondrial cytochrome c oxidase and F_1_F_0_-ATPase dysfunction in peppers (*Capsicum annuum* L.) with cytoplasmic male sterility and its association with *orf507* and *Ψatp6-2* genes. Int. J. Mol. Sci.

[23] Li J.J., Pandeya D., Jo Y.D., Liu W.Y., Kang B.C. (2013). Reduced activity of ATP synthase in mitochondria causes cytoplasmic male sterility in chili pepper. Planta.

[24] Lolis E., Alber T., Davenport R.C., Rose D., Hartman F.C., Petsko G.A. (1990). Structure of yeast triosephosphate isomerase at 1.9-A resolution. Biochemistry.

[25] Deng M.H., Wen J.F., Huo J.L., Zhu H.S., Dai X.Z., Zhang Z.Q., Zhou H., Zou X.X. (2012). Relationship of metabolism of reactive oxygen species with cytoplasmic male sterility in pepper (*Capsicum annuum* L.). Sci. Hortic.

[26] Raghavendra A.S., Padmasree K. (2003). Beneficial interactions of mitochondrial metabolism with photosynthetic carbon assimilation. Trends Plant Sci.

[27] Gilmore A.M., Bjorkman O. (1995). Temperature-sensitive coupling and uncoupling of ATPase-mediated, nonradiative energy dissipation-similarities between chloroplast sand leaves. Planta.

[28] Kromer S. (1995). Respiration during photosynthesis. Annu. Rev. Plant Physiol.

[29] Sabar M., de Paepe R., de Kouchkovsky Y. (2000). Complex I impairment, respiratory compensations, and photosynthetic decrease in nuclear and mitochondrial male sterile mutants of *Nicotiana sylvestris*. Plant Physiol.

[30] Dutilleul C., Driscoll S., Cornic G., de Paepe R., Foyer C.H., Noctor G. (2003). Functional mitochondrial complex I is required by tobacco leaves for optimal photosynthetic performance in photo respiratory conditions and during transients. Plant Physiol.

[31] Vaughn K.C., Lax A.R., Duke S.O. (1988). Polyphenol oxidase: The chloroplast oxidase with no established function. Physiol. Plant.

[32] Tingey S.V., Tsai F.Y., Edwards J.W., Walker E.L., Coruzzi G.M. (1988). Chloroplast and cytosolic glutamine synthetase are encoded by homologous nuclear genes which are differentially expressed *in vivo*. J. Biol. Chem..

[33] Moffatt B.A., Stevens Y.Y., Allen M.S., Snider J.D., Pereira L.A., Todorova M.I., Summers P.S., Weretilnyk E.A., Martin-McCaffrey L., Wagner C. (2002). Adenosine kinase deficiency is associated with developmental abnormalities and reduced transmethylation. Plant Physiol.

[34] Sun Z.J., Xu J.Q., Song M., Wei J.G., Tang Q.L., Wang Z.M., Wang X.J. (2012). Molecular cloning and expression analysis of annexingene from cabbage. Acta Hortic. Sin.

[35] Hause B., Stenzel I., Miersch O., Maucher H., Kramell R., Ziegler J., Wasternack C. (2000). Tissue-specific oxylipin signature of tomato flowers: Allene oxide cyclase is highly expressed in distinct flower organs and vascular bundles. Plant J.

[36] Browse J. (2009). Jasmonate passes muster: A receptor and targets for the defense hormone. Annu. Rev. Plant Biol.

[37] Stumpe M., Göbel C., Faltin B., Beike A.K., Hause B., Himmelsbach K., Bode J., Kramell R., Wasternack C., Frank W. (2010). The moss *Physcomitrella* patens contains cyclopentenones but no jasmonates: Mutations in allene oxide cyclase lead to reduced fertility and altered sporophyte morphology. New Phytol.

[38] Shen H.L., Jiang J.Z., Wang Z.Y., Geng S.S. (1994). Studies on the breeding and inheritance of male sterile lines of pepper (*Capsicum annuum*). Acta Agric. Univ. Pekin.

[39] Leja M., Mareczeka A., Benb J. (2003). Antioxidant properties of two apple cultivars during long-term storage. Food Chem.

[40] Wei J., Kenji T. (1995). Post-harvest changes of EFE activity, ACC and polyamine contents in cortex and pith of pear fruits and its relation to ethylene production. Acta Phytophisiol. Sin.

